# Annotations

**Published:** 1896-03-14

**Authors:** 


### Annotations.

### The Secrets of the Savoury Sausage.

In the meat trade truth is sometimes not only "stranger than fiction," but it is also much less delightful to the imagination. A case was brought before the Common Serjeant at the County of London Sessions the other day which for sheer disgustingness has, let us hope, few parallels. Here is an outline of the story. A farmer near Glastonbury had a cow which broke a bone, and which in consequence wasted away, and in the long run was shot. The carcase was then sold for seven shillings to a man named Chivers. The real value of it, if the cow had been healthy, would have been, not seven shillings, but twelve pounds. Chivers promptly labelled the putrefying mass "Cats' meat," and consigned it to John Harrington, meat salesman, who was then carrying on business in partnership with a man named Hopwood, in Charterhouse Street. Harrington did not, however, sell the cow's carcase to a cats' meat man, but to a sausage and brawn manufacturer at Bow, of the name of Frederick John Robinson. Robinson appears to have been proceeding with the work of converting the body of the diseased animal into sausages and brawn, when it was pounced upon by a sanitary inspector. This is the description of the stuff as it was found in the sausagemaker's establishment: it was "discoloured, slimy, devoid of fat, stinking, and utterly unfit for human food." Horrible! It is enough to make the mere name of "sausages" hateful to the average clean person. That, however, is not by any means the worst part of the business. Meat, in the course of putrefaction, is converted into "ptomaines," some of which are among the deadliest poisons known. Those poisons, instead of being destroyed by such cooking, or rather heating, as sausages usually undergo, are often greatly intensified in deadliness thereby. If the "slimy" and "stinking" carcase of the Glastonbury farmer's cow had not been impounded at Robinson's sausage shop, it might have caused the painful and distressing deaths of scores of unoffending persons, who would probably never have known what was the matter with them. Robinson was fined £50, which was a ridiculously small punishment under the circumstances. But Harrington, the meat salesman, we are glad to see, was sent to prison, with hard labour, for three months. The very urgent moral is, eat no sausages at all, unless the butcher who prepares and sells them has a reputation which is quite above suspicion. Why do not modern housekeepers, as their grandmothers used to do, have their sausages prepared at home by their own cooks?

### Typhoid and Tenancy—Another Legal Decision.

Legal precedents are gradually shaping the law in regard to tenancy and the special diseases arising from it in accordance with the principles of righteousness and common sense. Last week, in the case of Beadell v. Daw, before Mr. Justice Day and a common jury, Mr. Beadell, claiming damages for the death of his son from typhoid fever in consequence of the insanitary condition of the "flat" of which he had been tenant, was awarded £100; and a counter claim for rent made by the defendant Daw was disallowed, so that in each instance the victim of bad drainage won his case, with costs. The facts were simple. Mr. Beadell took an unfurnished flat of Mr. Daw through two persons, both of whom seem to have acted formally or informally as Daw's agents. Mr. Calvert, the first agent, assured Mr. Beadell that the drainage of the flat was what it ought to be; Mr. Hawkins, the second agent, seems to have confirmed the statement. The drainage proved to be old-fashioned and bad, and, in consequence, Mr. Beadell's son took typhoid fever and died. Some little time afterwards Mr. Beadell himself, and also his wife and daughter, were attacked by typhoid; but they all recovered. The doctor in attendance, having clearly no other course open to him, advised that the Beadells should leave the house, which accordingly they did as speedily as possible. Acting under legal advice, they gave notice to the landlord of what they had done, declined to pay the rent of the house during the typhoid period, and claimed damages, which, as already stated, were awarded to the amount of £100. The landlord, on his part, denied that his first agent had any right to warrant the wholesomeness of the drainage, and put in the second agent to deny that he had given any warranty, or, indeed, that any had been asked for. The points of importance to be noted are that no written warranty was given at all, and that a verbal warranty was denied. Notwithstanding both these circumstances, the judge and the jury, taking a common-sense view of the case, felt sure, not only that a warranty of sound drainage had been asked for, but also that it had been forthcoming, and judgment was given accordingly. We are aware that judgments have been given which have not been so satisfactory for tenants as this. Nevertheless, it is becoming more and more evident that evasions and the avoidance of written warranties will not in future be held to justify landlords in letting insanitary houses. In every case, a new tenant should obtain a written statement as to sanitary condition, but if he asks for no further warranty than the mere "spoken word," he should be prepared with witnesses.

### The New Photography in Court.

An interesting and novel case, in which the "X" rays practically decided the point, was tried by Mr. Justice Hawkins and a special jury at Nottingham the other day. Miss Ffolliott, a burlesque and comedy actress, whilst carrying out an engagement at a Nottingham theatre early in September last, was the subject of an accident. After the first act, having to go and change her dress, she fell on the staircase leading to the dressing-room and injured her foot. Miss Ffolliott remained in bed for nearly a month, and at the end of that time was still unable to resume her avocation. Then, by the advice of Dr. Frankish, she was sent to University College Hospital, where both her feet were photographed by the "X" rays. The negatives taken were shown in Court, and the difference between the two was convincingly demonstrated to the judge and jury. There was a definite displacement of the cuboid bone of the left foot, which showed at once both the nature and the measure of the injury. No further argument on the point was needed on either side, and the only defence, therefore, was a charge of contributory carelessness against Miss Ffolliott. Those medical men who are accustomed to dealing with "accident claims"—and such claims are now very numerous—will perceive how great a service the new photography may render to truth and right in difficult and doubtful cases. If the whole osseous system, including the spine, can be portrayed distinctly on the negative, much shameful perjury on the part of a certain class of claimants, and many discreditable contradictions among medical experts will be avoided. The case is a distinct triumph for science, and shows how plain fact is now furnished with a novel and successful means of vindicating itself with unerring certainty against opponents of every class.

